# Healthcare resource allocation decisions and non-emergency treatments in the aftermath of Covid-19 pandemic. How should children with chronic illness feature in prioritisation processes?

**DOI:** 10.12688/wellcomeopenres.19571.1

**Published:** 2023-09-01

**Authors:** Sapfo Lignou, Mark Sheehan, Michael Parker, Ingrid Wolfe

**Affiliations:** 1 Ethox Centre, Nuffield Department of Population Health, University of Oxford, Oxford, England, UK; 2 Wellcome Centre for Ethics and Humanities, Nuffield Department of Population Health, University of Oxford, Oxford, England, UK; 3 Institute for Women and Children’s Health, King's College London, London, England, UK

**Keywords:** Children, chronic illness, medical complexities, healthcare prioritisation, ethical framework, equality of opportunity, NHS, resource allocation

## Abstract

Background: In the aftermath of the Coronavirus disease 2019 (Covid-19) pandemic, allocation of non-urgent medical interventions is a persistent ethical challenge as health systems currently face an unprecedented backlog of patients requiring treatment. Difficult decisions must be made that prioritise certain patients over others. Ethical resource allocation requires that the needs of all patients are considered properly, but at present there is no guidance that can help support such decision-making which explicitly considers the needs of children with chronic and complex conditions.

Methods: This paper reviews the NHS guidance for priorities and operational planning and examines how the needs of children with chronic illness are addressed in NHS objectives for restoring services and meeting elective care demands.

Results: The usual criteria for prioritisation featured in the NHS guidance fail to account for the distinct needs of children with chronic illnesses and fail to match more general considerations of what constitutes fair resource allocation decisions. To address this issue, two considerations, namely 'protecting age-related opportunity' and 'recognising complexity of care,' are proposed as additions to the existing approach.

Conclusion: By providing a broader conception of needs, these criteria address inefficiencies of the current guidance and relevant ethical frameworks and help to embed a currently missing children-related ethical approach to healthcare policy making in general.

## Introduction

In the aftermath of the Coronavirus disease 2019 (Covid-19) pandemic, allocation of non-urgent medical interventions is a persistent ethical challenge. Elective services have now resumed, but due to reduced access to non-critical care during the pandemic and the significantly increasing number of patients with non-communicable conditions simultaneously requiring treatment, considerable strain is placed on health care systems. Tackling the elective backlog involves practical and clinical challenges for healthcare institutions and clinical practices. Such decisions also require complex ethical considerations that have a wide-ranging impact on different populations. As there are not enough resources to treat all patients in need, some will inevitably be denied the effective medical treatment they require.

Among those whose care has been significantly affected by the pandemic and its policies and who now must wait in queue for treatment is a large number of children with chronic illness ^1– 3^. Children with chronic illness and in particular those with medical complexities ( *e.g.* cystic fibrosis, sickle cell disease, and traumatic injuries) are considered a particularly vulnerable patient group with variable and often poor health outcomes due to systemic weaknesses ^4^. Paediatricians and child health organisations have argued that it is essential that high quality accessible healthcare is maintained and improved in the aftermath of the pandemic for this group ^5^. Health systems and their components and functions are interdependant and redirection of important resources (medical supplies, clinical staff, supplies and space) necessary to respond to increasing demand inevitably affects the standard of care for those children and their opportunities to receive the treatment they need. As healthcare systems deliver plans across elective services, including specialised services for adults and children ^6^, it is important to ensure that the needs of all patients, including children with chronic illness, are properly considered when decisions regarding whom to treat and which medical services to offer are made.

In this paper, we focus on the UK National Health System (NHS) and examine how the needs of children with chronic illness feature in NHS objectives for restoring services and reducing the COVID-related backlogs. ^1^ We review current criteria for prioritising non-urgent care in the NHS priorities and operational planning guidance 2011/2022 and 2022/2023 ^7^ and discuss each of those criteria separately. We argue that current guidance fails to provide a fair scope for rationing healthcare treatments for the paediatric population and in particular for children with chronic illness. To ensure that the distinct needs and vulnerabilities of those children are not neglected and improvements in children and young people’s (CYP) care and health outcomes are given proper weight in any deliberation about resource allocation in the aftermath of the pandemic, we suggest that two additional considerations are incorporated in current NHS guidance and related ethical frameworks: ‘protecting an age-relative equality of opportunity’ and ‘considering complexity of care’. We argue that these criteria address a currently missing children-related ethical dimension ^2^ in current healthcare policy objectives and, consequently, a more comphrehensive account of needs, necessary to guide fair decision-making in healthcare resource allocation. In the final section, we consider a number of objections associated with the implementation of our framework.

Our discussion is restricted to clinically necessary non-emergency services that are considered standard treatment by the NHS, with the aim of supporting the decision-making processes for commissioners and providers of healthcare.

## Methods

A document review approach was employed to review the current criteria for prioritising non-urgent care in the NHS priorities and operational planning guidance for 2011/2022 and 2022/2023. The relevant documents were thoroughly analysed to understand the specific criteria used for prioritisation. Each criterion was examined separately to assess its alignment with the distinct needs and vulnerabilities of children with complex conditions.

### The distinct needs of children with chronic illness and medical complexities

Before we discuss the NHS criteria for prioritisation, let us examine the reasons for which children with chronic and complex conditions present a distinct case for ethical consideration in healthcare prioritisation decisions by exploring their distinct needs and vulnerabilities.

These needs fall into two broad categories. The first category is associated with the nature of childhood specifically (and thus is in contrast to adult needs) and refers to children’s growth and developmental needs ^8^. The second category of needs is related to the nature of chronic illness. Children, like anyone who suffers from a chronic condition, have a range of illness-based needs characterised by frequent pain or discomfort and reduced quality of life for which planned and urgent healthcare is required ^9^. These with multiple morbidities or comorbidities, and in some cases multiple, distinct healthcare conditions have more complex needs for which a range of interventions and healthcare packages are necessary to manage each of the different sets of symptoms ^10^.

These two categories of need (those related to the nature of childhood and the nature of illness) interact with each other. The physical and psychosocial changes children with chronic illness experience during their development co-occur with their chronic or multiple health conditions from which they suffer and may lead to short-term or long-term challenges (such as disruption of the developmental process and psychosocial difficulties) ^11^. In adolescence in particular, chronic illness is reported to be associated with poorer social functioning and high rates of emotional and behavioural problems ( *e.g.* depression and anxiety) ^12, 13^. To respond to these problems, a range of targeted healthcare interventions and the provision of different services are necessary to improve disease management and to facilitate positive transitions to adult care ^14, 15^. In some cases, timely access to treatment may be crucial as the window for a positive effect of an intervention or treatment in children is smaller compared with adults ^16^. A child who, for instance, has to wait a long time for grommet surgery may suffer from deafness and impairments in language development which may lead to other serious non-clinical consequences, *e.g.* difficulties in learning and attending school, in participating in typical social interactions ^17^.

As the above example illustrates, the importance of healthcare does not only stem from its impact on an individual’s health but also on the effect it can have on the achievement of normal functioning and life opportunities ^18^. All patients with chronic conditions heavily rely on healthcare provisions to alleviate distressing symptoms so they can effectively go on living and have the ability to experience goods in life ( *e.g.* to work or attend school, socialise etc). But for the paediatric patients, healthcare provision is particularly important; it serves the immediate interests of a child with chronic illness and also their developmental and future interests ^19^ ( *e.g.* their ability to be or become independent and manage their own condition, their susceptibility to further disabilities or illnesses in adult life) and the range of opportunities that could be open to them in later life ^20^ ( *e.g.* their ability to pursue future careers).

### NHS criteria for restoring elective care and prioritising patients

One of the main current objectives of the NHS is to recover and restore elective clinical activity that that was lost due to the pandemic and to reduce long waits by significantly increasing the number of people that can be diagnosed, treated, and cared for in a timely way ^21^. Several strategic purposes are set out in the NHS guidance: improving outcomes in population health and healthcare, tackling inequalities in healthcare outcomes, experience and access, enhancing productivity and value for money, and supporting broader social and economic development. Aligned with these strategic objectives, four criteria can be identified in this document that are intented to provide an ethical basis for deciding which groups should have priority access to limited healthcare resources: ‘Prioritising those with those with longest waits’; ‘Prioritising the most clinically urgent needs; ‘Using the resources in order to produce the best overall outcomes’; and ‘Addressing inequalities in access, experience, and outcomes in healthcare’.

***Prioritising clinically urgent needs.*** When there is high demand and limited healthcare resources, not all medical needs can be met. A just allocation of different types of health care services requires that the competing claims of different patients with healthcare needs are taken into account and priority is given to those that are most justified ^22^. Clinical urgency is considered as the main criterion, based on which the importance of a need is assessed: according to current NHS guidance these patients, *e.g.*, for cancer and P1/P2 surgery, are prioritised for treatment. Clinical urgency is based on prognosis and current relieveable suffering. A patient who stands to lose more or suffer more has a stronger claim to the treatment ^23^. This criterion seeks to minimise harm during a time of pressure on the health system and it is particularly important for medical centres that serve the needs of large geographic catchment areas, for example through referral, and by providing specialist, rare, and network-based services. Patients are classified as those who urgently require treatment and those whose needs are less urgent and thus can be treated later or who are likely to recover without treatment ^24^.

Prioritising those with most clinically urgent needs reflects our moral intuition of the importance of saving those who are worse off (the sickest) before others who also stand to benefit from the same treatment ^25^. However, if this criterion is strictly applied, it deprioritises important categories of long-term care services and more effective use of resources. By focusing on how worse off someone is compared to others at the current time, this criterion disregards a less acutely ill person with progressive illness, who might suffer more later ^26, 27^ assuming that they will have an opportunity for treatment in the future ^28^. This problem is key for patients with chronic illness who are prone to pain and reduced quality of life, and for whom good quality of routine care is required.

The ‘most clinically urgent needs’ criterion, as presented in the NHS guidance, is particularly problematic for children with chronic illness as it apparently discounts effects of progressive illness on a child’s developmental needs and long-term impact on their wellbeing. Athough assessing the clinical risk for those on waiting lists is relevant for prioritisation, no specific reference is made for the paediatric population, despite the fact that many treatments are age and developmental stage critical ^29^.

***Prioritising those with longest waiting times.*** Based on the NHS guidance, how long someone has waited to be treated matters for fair prioritisation ^30^. This criterion is distinct from clinical urgency because it need not depend on the severity of the need (though when considering it in combination with the criterion above, differences in clinical need should clearly be weighed against differences in waiting time). If two patients are, other things considered, clinically similar, the patient who has waited longer has a greater claim to be prioritised.

While this criterion is perfectly plausible and certainly captures an important feature of fariness when it comes to waiting lists, it clearly also fails to allow for any special case for the needs of children with chronic and complex needs. The lack of a distinction between the paediatric and the adult population, despite their distinct needs, is clear in the guidance. With the exception of cancer and mental illness, no other considerations for patients with chronic and complex conditions are made, despite the severity of their needs and their great dependency on planned care. This criterion cannot help to address the omissions of the ‘urgent needs’ criterion mentioned above.

***Producing the overall best outcomes.*** An important aim of the NHS is to use resources in a most effective and efficient way ^31^. In order to meet this aim, prioritising the most clinically urgent needs or those with the longest waiting times cannot be the only criteria when deciding between patient groups with competing claims for treatment. Giving complete priority to those who are worse off without considering post-treatment prognosis would suggest prioritising patients even when only minor benefits can be achieved and even at high cost ^32^ which would have to forego doing more good for others ^33^.

A third criterion for prioritisation included in the NHS guidance that complements the ‘most urgent needs’ criterion focuses on an intervention’s value, *i.e.* how much benefit healthcare resources can provide. Allocation decisions based on capacity to benefit are arguably the dominant view of what justice requires in these contexts. This approach is often closely associated with the UK’s National Institute for Health and Clinical Excellence (NICE) in the form of cost per quality-adjusted life-years (QALYs) ^34^. Benefit is measured according to how many additional life years health care resource allocation can produce, multiplied by a percentage reflecting the quality of life to be experienced during those years ^35^. So, in the abstract, a decision between two patients who need the same treatment but differ in how long their lives can be prolonged will favour the person with the potential to receive the greatest benefit, *i.e.* live longer.

The cost-benefit or cost-effectiveness criterion is based on the rationale that having more and quality years of life is of greater value than having fewer, so prioritising patients with the aim of doing as much good as possible has intuitive appeal ^36^. This criterion informs the NHS plan to organise and deliver services in ways that “maximise productivity opportunities and secure the best possible outcomes for patients” ^37^.

This criterion broadly favours children compared to adults in treatment prioritisation decisions because of their greater life expectancies ^38^: a child who receives effective treatment will have more life years than an adult in a similar situation, and so the cost per life-year will be lower. However, ethical problems with cost-benefit calculations and allocation decisions, which have been extensively discussed in the literature when it comes to unfair distribution ^39^, are also relevant in the case of many children with underlying illness and disabilities that shorten their life span ^40^. This criterion also favours those whose conditions need fewer resources for treatment (in terms of money, time, staff) and consequently inherently discriminates against a smaller group of around 80,000 CYP with multifaceted needs who require health care from a diverse group of health practitioners and a range of different providers ^41^.

When it is overused, an approach that favours distributing resources in order to achieve the overall best outcome, departs from judgments many people would make about what is fair. Even when there is overwhelming demand, we tend to consider that patients who have only lived a short life should not be disregarded in prioritisation decisions irrespective of their survival-limiting comorbidities, and the frequency or complexity of their needs ^42^. Research on people’s preferences in healthcare resource allocation choices align with this judgment ^43^. According to evidence, many people are willing to sacrifice overall health gains to favour a young patient with poor lifetime prospects ^44, 45^. A decision not to expend substantial resources on a patient in order to avoid compromising the resources needed by others is reasonable under resource scarcity. But for such decisions to be fair, differences between potential candidates for treatment, such as the severity of their needs ^46^, the number of years they have so far lived ^47^ or the life opportunities they haven’t yet enjoyed ^48^, are important to be taken into account.

***Tackling inequalities in outcomes, experience, and access to healthcare.*** Whereas some of the criteria mentioned above justify prioritisation decisions that derive the greatest benefit ( *e.g.* saving most lives or treating more people), a different criterion included in the guidance aims to give a broader range of individuals an opportunity for receiving a benefit. “ {We} continue to develop our approach to population health management, prevent ill health and address health inequalities – using data and analytics to redesign care pathways and measure outcomes *with a focus on improving access and health equity for underserved communities*” ^49^. Based on this statement, the efficient use of scarce healthcare resources and the improvement of population health as a whole are not the only objectives of NHS policy; decision-makers should also be concerned about how healthcare benefits are distributed.

This additional consideration in NHS guidance takes into account relational and relative differences between patients that arise for historically disadvantaged or burdened groups. Deprived populations and certain ethnic minority groups have been shown to have poorer access to health care prior to the pandemic and experienced poorer health compared to the rest of the population ^50, 51^. There is also evidence that unequal access to healthcare and social injustices across populations can compromise the health of those most vulnerable in times of crisis ^52, 53^. With the aim of responding to these problems, the NHS has set one of its main priorities during the Covid recovery “to create a system of health and care that tackles both the health inequalities and the other *weaknesses that covid-19 has exposed*… with a *particular focus on analysis of waiting times by ethnicity and deprivation”* ^54^.

Addressing inequalities in access and experience of healthcare is important to improve health and healthcare outcomes for disadvantaged population groups. Given that the biggest increases in waiting lists are in the areas of greater deprivation ^55^, efforts to eliminate healthcare disparities may help eliminate existing health inequities exacerbated by the pandemic ^56^. Additionally, by accounting for ethnicity and deprivation in the analysis of waiting times, the NHS may help decrease the likelihood of harm to those already disadvantaged due to delayed or omitted treatment. However, inequality in access to, and experience of healthcare may represent a separate ethical reason to give a patient special consideration ^57^. The aim to address inequalities in access and experience of healthcare may also suggest that considerations beyond urgency and the likelihood of benefit should be taken into account in decisions regarding just distribution of treatments for everyone who is waiting, such as the importance of equalising social opportunities in accessing and receiving care.

In theory, many children with chronic and complex conditions would satisfy conditions for healthcare prioritisation based on the above considerations. Due to inefficiencies of the hierarchical referral model of primary to secondary care and the poor communication between different providers and services required for their multifaceted care, children with chronic and complex needs traditionally face health inequities with variable and sometimes poor health outcomes ^58^. During the pandemic, longstanding health inequities and healthcare inequalities were compounded by social disadvantages ( *e.g.*, financial or language barriers in accessing remote care) ^59^ and further diminished the care of many of these children ^60^.

Moreover, during the pandemic, the needs of many children with complex and long-term conditions have changed; some will have outgrown their equipment and others may have faced further challenges as a result of missing their usual developmental support and access to therapies ^61– 63^. To meet any new and more complex care needs, multiple interventions and complex packages of care may be necessary ^64^. Among adolescents targeted and complex support during the transitional period to adult healthcare may be needed. This vulnerable group whose needs have often been neglected is particularly important to be prioritised ^65^.

In the NHS plans for the recovery of elective care, however, most attention has been placed on reducing long waits and increasing the number of patients that can be treated. The amount of healthcare resources required for care for the groups of children described here is therefore likely to be disregarded. Neglecting the distinct needs and care requirements of children with chronic and complex needs can further exacerbate pre-existing unfair health and healthcare inequalities for this population group instead of addressing them. This is particularly problematic for children with medical complexities and their families as they often face care and financial challenges and barriers in accessing care ^66^.

Unmet needs can have severe clinical and non-clinical consequences for children with complex needs which can also be associated with negative behavioral health outcomes such as non-adherence to medication in adolescence ^67, 68^, delays in the feeding skills, speech or skeletal development in younger children and serious impairments that may significantly restrict their life opportunities ^69^. In order for the NHS to address unfair health outcomes, and for healthcare to be inclusive, successful, and resources fairly distributed, the prior disadvantage of children with chronic and complex conditions due to systemic inefficiencies and risks associated with their unmet needs should be clearly acknowledged and addressed.

## Additional ethical considerations for patient prioritisation

### ‘Fair share of resources’ in the aftermath of the pandemic: Deciding between patients with different medical needs and in different stages of life

When there is scarcity of healthcare resources and different population groups are in competition with one another for treatment, a principled way that takes into account morally relevant differences between them is essential to guide healthcare resource allocation decisions. Fairness demands that all needs are taken into account, but we have argued above that current NHS guidance considers a restricted scope of needs. By relying on criteria which disregard important aspects of the needs of chronically ill patients ( *e.g.*, poor quality of life, medical complexities) and of the paediatric population ( *e.g.*, developmental skills) children with chronic illness, a large population group with various but distinct vulnerabilities is excluded from considerations based on which healthcare prioritisation decisions are made.

An essential step towards recognising an equal standing of children with chronic illness in healthcare resource allocation approaches to justice is to acknowledge their distinct clinical care requirements and in particular those they have in virtue of being children. To incorporate these considerations to the existing NHS guidance, two ethical criteria are described below ‘protecting equality of age-relative opportunity’ and ‘acknowledging complexity of care’. These criteria offer a currently neglected children-related ethical perspective in healthcare resource allocation but their application is not restricted to this population group. These added considerations also capture morally relevant differences in the needs of different patient and population groups and thus provide a more comprehensive approach to the complexity and contextuality of the elective care backlog.

### Protecting age-relative equality of opportunity

With the exception of mental health services (where there is the most pressing paediatric backlog) and the care of autistic children, no other mention of the paediatric healthcare backlog is made in the NHS guidance. The avoidance of drawing a distinction between adult and paediatric patients in healthcare prioritisation decisions usually relies on the assumption that children and adults should be treated equally in order to be treated as equals ^70^. Yet the distinct needs of the paediatric population, and in particular of children with chronic and complex conditions, suggest that this approach is based on a problematic conception of equality among potential candidates for treatment. 

Norman Daniels has developed an account of just health that includes stages of life in the scope of healthcare resource distribution and can therefore provide us with some guidance on this issue. He developed the view of ‘age-relative normal opportunity range’ which is sensitive to the fact that lives have phases in which different general goals and tasks are central ^71^. Healthcare systems, he argues, should allocate resources required in each life stage to the greatest extent possible justly so that each patient has the ability to experience the goods of certain stages of life ^72^.

Based on Daniel’s account, we propose a new criterion ‘protecting age relative equality of opportunity’ to complement the existing needs-based criteria both in the NHS guidance and other related ethical frameworks ^73^. By relying on the view that in patient prioritisation decisions not only health gains, but also opportunities to health gains should matter, this criterion suggests an age related needs-based approach of justice that aims to give everyone a fair equal opportunity to receive treatment. On this view, stages of life classification should matter for healthcare resource allocation. ^3^ This is because meeting health needs is not only important to alleviate a patient from pain and discomfort, but also to enable them to have and to lead a normal life with a normal set of life opportunities at different stages of life. ^4^

In contrast to the other criteria, this criterion directly captures the claims of children with chronic and complex needs to fair and equal consideration. By introducing the idea that everyone should have an opportunity to receive care necessary for their distinct age-related or ‘stage of life’ needs, it draws attention to the value of timely-critical care on the developmental skills of a child with chronic and complex conditions. This criterion also highlights that children with chronic conditions deserve healthcare services and benefits derived from them at least as much as children with better quality of life and fewer complexities, even if the amount of benefit they will receive after treatment will be lower compared to other patients. ^5^ Children with chronic illness *qua children* ought to be given a fair opportunity for healthcare necessary for their immediate, developmental and future interests ^74^ in order to experience, to the extend that is possible, the goods appropriate for their stage of life ( *e.g.* to attend school, enjoy time with family and friends). For this to be acheived, distinct consideration should be given regarding their care ( *e.g.* the impact of delaying treatement essential for their development and flourishment) to ensure an inclusive and fair process to decision making in the recovery of health services.

Yet, the needs of children with chronic and complex conditions are but one consideration that bears on the justice of health care distribution. Recognising that in each stage of life people have different needs and vulnerabilities, and in these stages different opportunities should be afforded, this criterion requires that all age groups needs (such as the distinct needs of the elderly population) should also be considered in healthcare prioritisation decisions to inform fair allocation processes. ‘Protecting age-relative equality of opportunity’ can thus be considered as a just healthcare allocation criterion ^6^ that accommodates morally relevant differences when choosing between the competing claims of patients across the age spectrum requiring care. It can therefore be used as a crude measure of the priority we should give to health services by taking into account patient needs and their importance at different life stages.

### Acknowledging complexity of care

Children with chronic illness, and in particular those with complex needs, provide a clear example of a group of patients whose clinical circumstances are significantly different to those of the population of patients for whom the NHS recommendation has been made. By focussing on numerical targets for success, the NHS policy has the effect of considering elective backlog as a single treatment or intervention to take off the list ^75^ and thus ignores some groups of patients whose severity and complexity of need were increased during the pandemic ^76^. 

To address this ‘complexity’ problem, we argue that a new criterion ‘acknowledging complexity of care’ should also be included in current NHS guidance for healthcare prioritisation that can enable the needs of patients with medical complexities to be equally considered. By focusing on equal opportunities in receiving healthcare rather than on benefit, this criterion suggests that a healthcare system should fairly allocate resources required for the complex needs of a patient even if the amount of benefit they will receive after treatment will be less compared to other patients ( *e.g.* even if some patients would enjoy a healthy life for a longer time).

This criterion also acknowledges that fair opportunities to receive care according to clinical needs does not require distributing the same amount of care to everyone. In order for a child with complex medical needs to effectively operate in the world, more healthcare resources may need to be allocated to them to manage each of their different sets of symptoms and their collective impact on their wellbeing. The ‘acknowledging complexity of care’ criterion then avoids some of the implications of the outcome-oriented criteria in decision-making and in particular the aggregation problem that systematically discriminates against this population group.

This criterion does not only concern children with complex needs. Decision-making about resource allocation is poorly equipped to deal with the complexity of the healthcare provision. This criterion enables healthcare prioritisation decisions to reflect broadly on healthcare complexities in an ethically robust way by, for instance, focusing broadly on across care delivery rather than single interventions. To ensure that policy makers are able to make informed, ethical decisions about resource allocation in the post-pandemic healthcare setting, the complex range of care required for patients with complex and multifaced needs should not be disregarded.

## Practical and contextual implications of this framework

In this paper, we have suggested the addition of two criteria to current NHS guidance for the development of treatment prioritisation protocols. However, in our analysis we have not suggested or prejudged the weight that any one criterion should be given, nor have we implied that all should be given equal weight. ^7^ Several factors may alter how decisions are made and how these criteria should be weighed against each other or be ordered lexically ( *e.g.* how to balance priority to the most urgent needs against maximising efficiency).

Decision-makers must consider each intervention and health service type uniquely and on the basis of the capacity of the service, the magnitude and nature of the clinical need, the severity of the problem of unmet demand ^77^. Due to the heterogeneity of hospitals (general hospitals *versus* children’s hospitals) and to differences in management of resources ( *e.g.* boundaries between paediatric and adult patients when sharing resources) there may be significant variation in the plans for resource distribution and allocation protocols. Providing guidance on how every medical service should approach prioritisation would therefore likely not be possible. 

Finally, and most importantly, we should note that these decisions require tradeoffs between different values ( *e.g.* between efficiency and fairness) for which there is no broad consensus on how to proceed ( *e.g.* should we prioritise ability to benefit over equal chances to receive care?). Decision-makers, clinicians and patient communities would disagree how specific trade-offs should be made, how much obligation we have to correct children’s impaired opportunities especially when costs are high ( *e.g.* very high demand for an intervention), whose needs should be deprioritised in order to enhance the opportunities of children with chronic conditions to receive care, and others. As Daniels notes, these ‘unsolved rationing’ problems reveal pervasive ethical disagreement ^78^.

Since such disagreements are reasonable, this framework alone cannot lead to resolution but should be supplemented by a “fair deliberative process” ^79^ to be legitimate. Those responsible for healthcare resource allocation decisions should have in place a fair and consistent mechanism for allocating resources and be able to demonstrate that all the relevant factors (such as those mentioned above) have been considered, as required by the NHS constitution (Principle 2A) ^80^. With recognition of the need for standardisation and transparency in prioritisation protocols, the degree of priority given to children with chronic and complex illness should be made explicit, with an ethical justification provided ( *e.g.*, how the suggested framework has generated a specific policy, and how it has been applied to the patients’ particular circumstances). Further work is needed to consider how this should be done, and given that there will be disagreement, transparent and fair processes including public involvement are essential.

Our framework, with its additions to current NHS guidance, is not intended to provide ready-made solutions to complex resource allocation ( *e.g.* how much weigh should be given to complexity when two patients suffer from equally painful conditions). Nevertheless, it is intended to be implementable and practically useful. First, it identifies important gaps in current, “in use” guidance and can inform existing resource allocation approaches. Second, the underlying ethical challenges related to meeting the needs of children with chronic conditions are common across different health and care systems. It is thus important that the processes themselves are able to consider with the degree of complexity required of them ^81^, even if it may not always be possible to fully meet those childern’s healthcare needs. Third, this framework provides a clear set of considerations and reasons that can be adapted for different processes and policy contexts in the light of evolving contexts. It can therefore function as a decision-support tool for authorities in primary care and general hospitals, when planning next steps for local services, and as such can be applicable to all UK healthcare institutions. Finally, our framework helps support a better understanding of the distinct needs of children with chronic and complex needs which is not only valuable for the purposes of NHS decision making in this context but also in providing the basis for a more comprehensive understanding of children’s equality and fair policy making in other contexts.

## Conclusion

The aim of this paper has been to address an increasingly pressing practical problem of justly allocating healthcare resources between different groups in the aftermath of the pandemic and in the face of significant waiting lists for treatment. Our focus has been on children with complex conditions, a vulnerable group that we argue has been systematically disadvantaged in policy considerations. We claimed that current NHS approach does not succeed in capturing their distinct needs. We suggested that two new criteria ‘protecting age-related equality of opportunity’ and ‘acknowledging complexity of care’ should complement the exiting ones in order to support decisions-makers to make decisions that are consistent, fair and inclusive to the needs of children with chronic conditions. We also considered broader implications of our suggested framework for health care resource allocation decisions in general. Though a fuller development of this framework is required to assess the application and practical considerations of our account, we hope that this work brings a better theoretical understanding closer to the practical changes faced in resource allocation decision making context in the aftermath of the pandemic and beyond.

## Data Availability

No data are associated with this article.

## Footnotes

^1^ It is important, however, to note that the decision-making processes are different in the different nations

^2^ We suggest these criteria in the specific context of children but, as we later discuss, their use is not restricted to them

^3^ As we discuss later, an important and difficult question is how much they should matter

^4^ There are important parallels here with the capability approach

^5^ This however does not suggest that the calculation should always go in their favour

^6^ Age is a clinical measure but decisions regarding prioritisation of patients cannot be based purely on clinical facts. Our approach considers both clinical and value-laden criteria for allocation and ethical judgments.

^7^ We do not, for instance, suggest explicit age-based prioritization or specify the weight to be given to complexity. Instead, both of these criteria should be considered as inputs for a discussion about resource allocation, rather than being treated as algorithms for making decisions.
