# Supplementary material for: Healthcare resource allocation decisions and non-emergency treatments in the aftermath of Covid-19 pandemic. How should children with chronic illness feature in prioritisation processes?
Source: Wellcome Open Res. 2024 Jan 16;8:385. Originally published 2023 Sep 1. [Version 2] doi: 10.12688/wellcomeopenres.19571.2 (PMC10835102; doi:10.12688/wellcomeopenres.19571.2)
Supplement: Peer review history

# Reviewer report: Reviewer response for version 2

**DOI:** 10.21956/wellcomeopenres.23046.r72692

**Published:** 2024-02-01

**Authors:** Richard Ashcroft

**Affiliations:** 1 School of Law, City University of London, London, England, UK

I am happy that the authors have responded appropriately to my review.

Is the work clearly and accurately presented and does it cite the current literature?

Yes

If applicable, is the statistical analysis and its interpretation appropriate?

Not applicable

Are all the source data underlying the results available to ensure full reproducibility?

No source data required

Is the study design appropriate and is the work technically sound?

Yes

Are the conclusions drawn adequately supported by the results?

Partly

Are sufficient details of methods and analysis provided to allow replication by others?

Yes

Reviewer Expertise:

Bioethics

I confirm that I have read this submission and believe that I have an appropriate level of expertise to confirm that it is of an acceptable scientific standard.

# Reviewer report: Reviewer response for version 1

**DOI:** 10.21956/wellcomeopenres.21681.r66488

**Published:** 2023-10-26

**Authors:** Richard Ashcroft

**Affiliations:** 1 School of Law, City University of London, London, England, UK

This is an interesting paper, presenting an analysis of how prioritisation criteria in recent NHS policies (2021/2 and 2022/3) fail adequately to allow for the specific needs of children with chronic illnesses and complex care needs. The paper goes on to present two criteria which could be added to current prioritisation criteria which would remedy this failure, in at least allowing those specific needs to be considered in prioritisation debates.

 The paper does a good job in demonstrating that the needs of children with chronic illness have special features to do with developmental needs, and the often complex care needs that go along with childhood illness. The argument from there is less clear. Implicit in the argument would appear to be a further criterion, which is that the needs of children, as children, have a certain weight. Certainly practical resource allocation in the NHS does seem to place weight on the special value we place on children's lives, during at least early childhood (hence the relatively high investment in paediatric intensive care, and the willingness of the public to give additional resources through charitable giving to children's hospitals). Another criterion might be to do with the quasi-iatrogenic nature of some childhood chronic illness due to COVID-19 infection or due to the public health protection measures adopted to prevent the spread of COVID-19 and their impacts on children. We might dissect that further - both there is an element of restorative justice to put right (unintended) harms caused to children by NHS and public policy decisions relating ot COVID-19, and element of reparative justice to do with a recognition that children's needs were sometimes overlooked during those decision-making processes. The paper is relatively short, so perhaps there was not space for much more analysis, but i think it is important to get more precision here about the precise nature of the claims children with chronic illnesses make _because_ they are children, _because_ they have chronic illness, or _because_ of social and historical circumstances. And further, we need to acknowledge the influence of public/social values in acknowledging (or not acknowledging) these claims, which are not necessarily the same as moral values as derived from theoretical reflection. 

 A second issue to consider is the way principles are intended to function in priority setting here. Noting that the authors occasionally rely on "intuitions", and explcitly refer to the work of Norman Daniels, they are working with some version of Rawlsian distributive justice as the theoretical frame, and some version of reflective equilibrium as the theoretical deliberative model. This could be made more explicit. But within that theoretical approach principles don't have any implicit ranking structure and no principle trumps any other. They are all taken together and through deliberation on particular cases some coherence is meant to emerge. So the authors are free to introduce new principles (as they do here) and then deliberation establishes how they apply and how they relate to other principles. There are some constraints on introducing new principles, however. One is that each such principle should be conceptually coherent in its own terms. Another is that they neither merely restate other principles, or are in logical contradiction with other principles (coherence is impossible where there is logical inconsistency). The authors could do more to show that they have satisfied these requirements in respect of the two new principles they introduce. One concern I have is that "age-relative equality of opportunity" is that it is not clear whether the authors mean specifically (numerical) age, or something more like developmental stage. If the former, there are well known difficulties with using numerical age, or even life expectancy, as an allocation principle. Another concern is that "acknowledging complexity of care" is, I think, flawed in two ways. First is that it does not seem to guide priority setting: is "complexity" a criterion in addition to the conjoined claims of each element in a list of conditions or needs? Or is saying that a patient has complex needs simply to restate that "priority setting here is really hard"? Or is it the inverse of an intution that "complex needs" entails "low priority" (because its too costly to get any benefit or make any material difference) - that seems to be what is entailed by "priority to the worst off" and is in contradiction to what might be entailed by "maximising QALYs".

 Fourthly, are we to understand "children with chronic illness" as a group needing consideration in their own right, or are they a sort of test case which shows more general problems in our current priorisation criteria and processes?

 Finally, I note that the authors accept that some kind of public deliberative approach to priority setting is necessary and implied by their adoption of the Daniels-type methodology. All I would say to that is that in what one might consider ideal conditions for having a very focussed public debate about such questions during the COVID-19 pandemic, we did not do so, and its worth considering why not. (By we I mean in the UK, but arguably nowhere in the World did this happen, and indeed its arguable that "bioethics" went missing in action).

Is the work clearly and accurately presented and does it cite the current literature?

Yes

If applicable, is the statistical analysis and its interpretation appropriate?

Not applicable

Are all the source data underlying the results available to ensure full reproducibility?

No source data required

Is the study design appropriate and is the work technically sound?

Yes

Are the conclusions drawn adequately supported by the results?

Partly

Are sufficient details of methods and analysis provided to allow replication by others?

Yes

Reviewer Expertise:

Bioethics

I confirm that I have read this submission and believe that I have an appropriate level of expertise to confirm that it is of an acceptable scientific standard, however I have significant reservations, as outlined above.

# Reviewer report: Reviewer response for version 1

**DOI:** 10.21956/wellcomeopenres.21681.r66484

**Published:** 2023-09-29

**Authors:** Richard Hain

**Affiliations:** 1 Welsh Paediatric Palliative Care Network, NHS Wales Cardiff and Vale University Health Board, Cardiff, Wales, UK; 2 School of Medicine, University of Liverpool, Liverpool, England, UK

This is a piece of philosophical research in ethics. It addresses one of the ethical questions facing healthcare providers in the aftermath of the COVID pandemic; namely, how to decide who should be treated first in the face of an enormous backlog of patients with chronic conditions. 

 The authors note that the criteria for prioritising such patients in the UK's NHS are the degree of urgency, the length of time that patient has already waited, effectiveness of the delayed intervention relative to its cost, and the extent to which further delay would exacerbate existing healthcare inequities in society.

 The report's authors represent an argument that the nature of children and the chronic conditions from which children suffer is such that, to a greater or lesser extent, those criteria inevitably and systematically disadvantage children with chronic conditions. Furthermore, the authors argue that the criteria do not adequately take into account aspects of the nature of children that are relevant to existing criteria, such as the child's potential for more years of benefit compared with an adult and the highly complex care that a child with chronic illness typically needs.

 The authors propose two new criteria which they believe would address the disadvantage that children with chronic conditions currently face. They argue that the first, 'age-relative equality of opportunity', would give value to a child's nature as a being who is still developing and so will benefit from interventions well into the future. The second, 'acknowledging complexity of care', would give value to the wider burden on others that a child's chronic illness imposes.

 There is currently no coherent system of ethics that inherently accommodates the nature of children. In the face of that lacuna, the authors frame the argument clearly and compassionately in the context of current thinking, particularly Daniel's account of stage-related 'goods'. 

 Currently the authors take issue with each of the criteria in turn. I wonder if the flow would be clearer if the authors first set out the criteria as they currently exist without commenting on them, then set out their concerns, and finally propose their solution.

 The fundamental argument underlying this piece is one I strongly endorse. That is, the assertion that a system of medical ethics that is adult-normative, as most are, always risks undervaluing children and so underestimates their needs. This report offers an important and timely illustration of that broad principle, and of the need for an approach to ethics that evaluates children according to their own nature where it is meaningfully different from that of an adult.

 Adding a criterion that gives value to complexity, and the burden such complexity imposes on others, seems to me well-considered and practical. The criterion itself would apply equally to adults and children, and there are measures of complexity already available.

 Adding a criterion that gives value to potential future years of benefit seems to me more problematic:

1. It is a matter of judgment rather than something that can be measured
2. It would systematically give less priority to patients on the basis of how long they were likely to survive. It would mean, for example, giving less priority to elderly adult patients that to younger ones. In passing, since women live longer than me it could be argued (pedantically) that such a system would also introduce gender bias.
3. It would systematically give less priority to any child with a chronic condition that is expected to reduce their life span. That would include a large proportion of children with complex chronic conditions.  

I wonder if something could be achieved by expressing waiting time as a proportion of the patient's life to date, rather than in months and years ? So that a child of 10 waiting a year would be prioritised in the same way as a woman of 60 waiting 6 years ? Or some variation along those lines. That would be in accordance with some theories of subjective perception of time. Outside childhood, however, that would introduce differential valuations according to a patient's objective age which I think most people would find unacceptable. Perhaps that could be introduced as a criterion specifically for children ?

Is the work clearly and accurately presented and does it cite the current literature?

Yes

If applicable, is the statistical analysis and its interpretation appropriate?

Not applicable

Are all the source data underlying the results available to ensure full reproducibility?

No source data required

Is the study design appropriate and is the work technically sound?

Yes

Are the conclusions drawn adequately supported by the results?

Yes

Are sufficient details of methods and analysis provided to allow replication by others?

Yes

Reviewer Expertise:

Medical ethics in children (especially at the end of life)

I confirm that I have read this submission and believe that I have an appropriate level of expertise to confirm that it is of an acceptable scientific standard.
